# Gut microbiota composition and diversity before, during, and two months after rifamycin-based tuberculosis preventive therapy

**DOI:** 10.1038/s41598-023-44854-5

**Published:** 2023-11-02

**Authors:** Marie Nancy Séraphin, Julia Bellot, Emily Klann, Maria Ukhanova, Florence G. Saulsberry, Charles A. Peloquin, Volker Mai

**Affiliations:** 1 Department of Medicine, Division of Infectious Diseases and Global Medicine, College of Medicine, University of Florida, Gainesville, FL USA; 2 Emerging Pathogens Institute, University of Florida, Gainesville, FL USA; 3 Department of Epidemiology, College of Public Health and Health Professions and College of Medicine, University of Florida, Gainesville, FL USA; 4 Florida Department of Health in Alachua County, Disease Control Unit, Alachua County Health Department, Gainesville, FL USA; 5 Infectious Disease Pharmacokinetics Laboratory, College of Pharmacy, University of Florida, Gainesville, FL USA

**Keywords:** Tuberculosis, Epidemiology, Microbiome, Antimicrobials

## Abstract

Tuberculosis (TB) preventive therapy (TPT) is an effective strategy to eliminate TB in low-incidence settings. Shorter TPT regimens incorporating the antimicrobial class of rifamycins are designed to improve adherence and completion rates but carry the risk of modifications to the gut microbiota. We enrolled six subjects diagnosed with latent TB infection (LTBI) who accepted to initiate TPT. We also enrolled six healthy volunteers unexposed to the rifamycins. We profiled the gut microbiota using 16S rRNA amplicon sequencing (V1-V2 region) to document the immediate effect of rifamycin-based TPT on the gut microbiota composition and tracked recovery to baseline two months after TPT. Overall, TPT accounted for 17% of the variance in gut microbial community dissimilarity. This rifamycin-based TPT induced dysbiosis was characterized by a depletion of butyrate-producing taxa (Clostridium-XIVa and Roseburia) and expansion of potentially pathogenic taxa within the Firmicutes and Proteobacteria phyla. Recovery of the gut microbial composition was incomplete two months after TPT. Robust clinical studies are necessary to comprehensively catalogue TPT-induced gut microbiota dysbiosis to inform strategies to mitigate potential long-term sequelae of this important TB control intervention.

## Introduction

*Mycobacterium tuberculosis* (Mtb) is a persistent pathogen that continues to cause significant disease burden^1^. In 2021, an estimated 10.6 million people developed tuberculosis (TB) disease, and 1.6 million died from TB^1^. Upon exposure to Mtb, infected individuals either develop primary TB disease or latent TB infection (LTBI)^2^. A quarter of the world population is estimated to have LTBI, defined as clinically asymptomatic TB infection^2,3^. People diagnosed with LTBI have an estimated 10% lifetime risk of developing TB disease^4^. Children, healthcare workers, recent immigrants from high TB incidence countries, recent contacts of a TB case, and immune compromised individuals, such as people living with the human immunodeficiency virus (HIV) are at increased risk of progression to TB disease if diagnosed with LTBI^4,5^. These high-risk groups are thus prioritized for TB preventive therapy^5^. In the absence of a vaccine that prevents Mtb infection or progression to TB disease if infected, protracted therapy with a combination of antimicrobials is currently our most effective TB control approach^6^. 

In the United States (U.S.), diagnosis and treatment of LTBI in high-risk groups is a cornerstone of the national strategy to eliminate TB^7^. Shortening the duration of this TB prevention therapy (TPT) is instrumental to meeting the TB elimination goal of one TB case per million population^8^. The first available TPT regimen, a six to nine-month course of isoniazid (INH) administered daily continues to be prescribed, despite its low completion rate^9^. Shorter TPT regimens can increase treatment compliance as well as reduce the risk of adverse events associated with protracted therapy^10,11^. Various alternative shorter TPT regimens are currently approved for use, including regimens that combine INH with the antimicrobial class, rifamycin [rifampin (RIF) and rifapentine (RPT)]^12–14^. Currently, HIV uninfected individuals can complete TPT within twelve to sixteen weeks with either a weekly course of a combination of INH and RPT( 3HP), or daily RIF for four months (4R)^6^. A one-month regimen of INH and RPT has been shown to be efficacious in treating LTBI in people living with HIV (PLWH)^14^. These shorter combination TPT regimens are quickly being adopted as they have been associated with increased patient adherence and completion rates^11^. 

The antimicrobials used to treat TB infection, including INH, pyrazinamide, and ethambutol, primarily target mycobacterial species^15^. However, the rifamycins, which are a cornerstone of anti-TB short-course chemotherapy^16^,  may inhibit transcription in a broad spectrum of bacteria by binding to the beta subunit of RNA polymerases^17^. The rifamycins are extensively metabolized in the liver to their partially active 25-O-desacetyl metabolite that is eliminated in feces^15^. The rifamycins undergo enterohepatic recirculation, where the drug is reabsorbed from the intestines back into the bloodstream^18^. Enterohepatic recirculation prolongs the drug exposure time but may also increase the potentially negative effect of the rifamycins on the gut microbiota composition and function^19^. Indeed, early clinical investigations suggested that anti-TB treatment (ATT) triggers long-lasting gut microbiota changes^20^. Given the emerging literature on the role of the gut microbiota in Mtb susceptibility and the hypothesized contribution of ATT-induced gut dysbiosis on the host’s ability to fend off new Mtb challenge^21^, clinical studies are needed to inform the potential unintended consequence of an important public health intervention to prevent and potentially eliminate TB in low incidence settings^7^.

We conducted a prospective cohort study among individuals diagnosed with LTBI who accepted to initiate TPT with 3HP or 4R. We aimed to describe the immediate effect of the rifamycins on the gut microbiota and track recovery to baseline two months after therapy was completed. We also followed in parallel with the LTBI cohort, a healthy volunteer cohort we purposively recruited from a population assessed to be of low clinical suspicion for TB infection^5^. TB infection itself has been shown to modify the composition and function of the human microbiome^22,23^. In addition, the human microbiome is uniquely influenced by the host’s lifestyle choices throughout the lifecourse^24^. We included the healthy volunteer cohort to isolate the independent effect of the rifamycins on the gut microbiota of the LTBI volunteers. To do this, we contrasted the longitudinal changes in gut microbiota alpha and beta diversity observed following exposure to the rifamycins to changes we measured by prospectively profiling the gut microbiota of our healthy volunteers unexposed to the rifamycins.

## Methods

### Study setting and design

Our study was conducted at two Florida Department of Health TB (FDOH) clinics. Between February 2019 to October 2019, we enrolled 13 clients of the TB clinics diagnosed with LTBI who were candidates for TPT. Our LTBI volunteers had tested positive for TB infection, using either the tuberculin skin test (TST) or interferon gamma release assay (IGRA) test. To determine eligibility for TPT, patients underwent a medical exam and chest radiography to rule out TB disease^25^. The decision to offer treatment and the choice of regimen were per national guidelines for the treatment of LTBI^26^. Patients who accepted to initiate TPT with a rifamycin-based regimen (3HP or 4R) were eligible for study participation. We purposively recruited six healthy volunteers who met criteria for low-clinical suspicion for TB infection from our source population during the same period. The healthy volunteers self-reported a negative TST or IGRA test or were born in the U.S. without a history of travel to a TB endemic country. We limited study participation to subjects 15–65 years of age who were HIV-negative, non-diabetic, on therapy for any chronic condition, and without a history of antibiotic use in the month before enrollment. Study participation was for a maximum of six months and consisted of four stool collections completed at participants’ home on days 0, 30, 60 and 120 of TPT administered by FDOH TB clinics. Two LTBI subjects treated with 3HP by direct observation (DOT) collected stool samples at days 0,30,60, and 90 of therapy. Independent of the regimen prescribed, all LTBI subjects provided a final stool sample two months after completing TPT. The healthy volunteers were asked to collect stool samples at home on days 0, 30, 60, 120, and 180. The 30-day window for follow-up stool samples collection from the LTBI cohort was calculated from the date therapy was initiated. We calculated the 30-day stool collection window from the date healthy volunteers collected their baseline stool sample, ± 7 days (Supplementary Table 1). Full details of study design and stool sample collections and 16S rRNA (V1-V2 region) 2 × 250 amplicon sequencing using the Illumina Mi-Seq platform (Illumina®, San Diego, CA) are provided in supplementary materials, with additional details published here^27^.  

### Gut microbiota profiling

The paired-end sequencing reads were processed using the Callahan et al. analytic workflow^28^ implemented in RStudio^29^. Briefly, after trimming, filtering, and dereplication, raw reads were clustered into amplicon sequence variants (ASVs) using DADA2^30^ and assigned to a taxonomic class using the naïve Bayesian classifier method^31^ and the Ribosomal Database Project (RDP) v16 training set^32^. We applied prevalence filtering to retain phyla detected in at least 5.0% of all samples. We filtered out unclassified reads at the phylum level.

### Statistical analysis

The microbial community diversity and composition was described using ASVs randomly resampled to 33,582 sequencing reads. Alpha diversity (Shannon, Chao1, and Faith’s Phylogenetic Diversity (PD) within and between cohorts was summarized using median and interquartile range. Wilcoxon signed-rank tests were used to test the hypothesis of no difference in median alpha diversity during and post TPT compared to pre-TPT sampling periods in LTBI and healthy volunteer cohorts separately. We assess the evidence for a significant difference between the distribution of the alpha diversity indices of LTBI and healthy volunteer samples over the study duration using Mann–Whitney U test statistics. We computed unweighted and weighted UniFrac distances to describe the evolutionary relationship between the gut microbiota composition at baseline compared to the 30-day snapshots during TPT and sixty days after TPT^33^. Similarities and dissimilarities between microbial communities were visualized by principal coordinate analysis (PCoA)^34^ and statistical support for clustering by sampling intervals (before, 30 days on TPT, and 60 days after TPT) and rifamycin exposure (3HP, 4R or control) were evaluated using a permutational multivariate analysis of variance (PERMANOVA), with 999 permutations^35^. All analyses were performed in RStudio^29^. P-value adjustments for multiple comparisons used the Benjamini-Hochberg (BH) procedure^36^.

### Ethical approval and consent to participate

The research described involves human participants, human material, and human data. All experiments were performed in accordance with the Declaration of Helsinki. The study protocol was reviewed and approved by the University of Florida (IRB201801385) and the Florida Department of Health (2018–057) Institutional Review Boards. Written informed consent for study participation was obtained directly from each participant prior to all study activities.

## Results

### Subjects characteristics

After removing LTBI participants with incomplete data/samples, we retained six LTBI volunteers in the analysis. Six healthy volunteers completed all study activities and provided stool samples in parallel with the LTBI participants on 4R. The baseline characteristics of study participants are shown in Table 1. The study participants were predominantly of non-Hispanic ethnicity. While equal numbers were of non-US birth origin, participants with LTBI were born in countries where TB is more prevalent, consistent with TB epidemiology in low incidence settings, such as the U.S.^37^. The median baseline weight when all study participants was considered is 149.5 pounds, with similar baseline weight observed in the LTBI and healthy volunteer cohorts (W = 11, *p* = 0.31). The median age of the LTBI cohort was 31.0 years compared to 35 years in the healthy volunteer cohort; although the two cohorts did not significantly differ in age (W = 15, *p* = 0.687). Within the LTBI cohort, volunteers’ age (W = 3, *p* = 0.806) or baseline weight (W = 6, *p* = 0.533) did not differ by TPT regiment.

**Table 1:** Number of stool samples analyzed stratified by cohort and key baseline characteristics of study participants.

Cohorts	Participants	Stool Samples	Female (%)	Age (median)	U.S.-born (%)	Hispanic (%)	Weight (median)*
All subjects	12	60	58.3	32.5	33.3	33.3	149.5
LTBI—3HP	2	10	50.0	31.0	50.0	–	172.0
LTBI—4R	4	20	100.0	38.0	75.0	0.75	135.5
Healthy	6	30	33.3	35.0	66.7	0.17	157.1

*LTBI volunteers were weighed by the TB clinic while healthy volunteers self-reported their weight at enrollment. Formal Mann–Whitney U test statistics suggest the cohorts do not differ significantly on age (W = 15, *p* = 0.687) or baseline weight (W = 11, *p* = 0.31), and within the LTBI cohort, volunteers’ age (W = 3, *p* = 0.806) or baseline weight (W = 6, *p* = 0.533) did not differ by rifamycin exposure.

We generated 6,407,447 16S rDNA sequencing reads with an average of 97,703 ± 20,189 reads per LTBI volunteer sample and 115,878 ± 31,784 reads per healthy volunteer sample. After trimming, filtering, dereplicating, and denoising, the reads were classified into 6333 ASVs. Finally, we randomly resampled to an even sequencing depth to retain 5,612 ASVs and all 60 samples for statistical analyses. Phylum relative abundance in LTBI (AL002–MD001) and healthy volunteer (HC001–HC006) cohorts at each of the five time points sampled is shown in Supplementary Fig. 1. The most prevalent phyla across all participant samples were *Firmicutes, Bacteroidetes*, *Actinobacteria,* and *Proteobacteria*. We observed the phylum *Tenericutes* at 5% prevalence in all samples collected from three of the LTBI cases (AL012, AL003, and MD001) but none of the healthy volunteer samples.

### Immediate effect of rifamycin-based Tuberculosis Preventive Therapy on the gut microbiota

**Figure 1:**
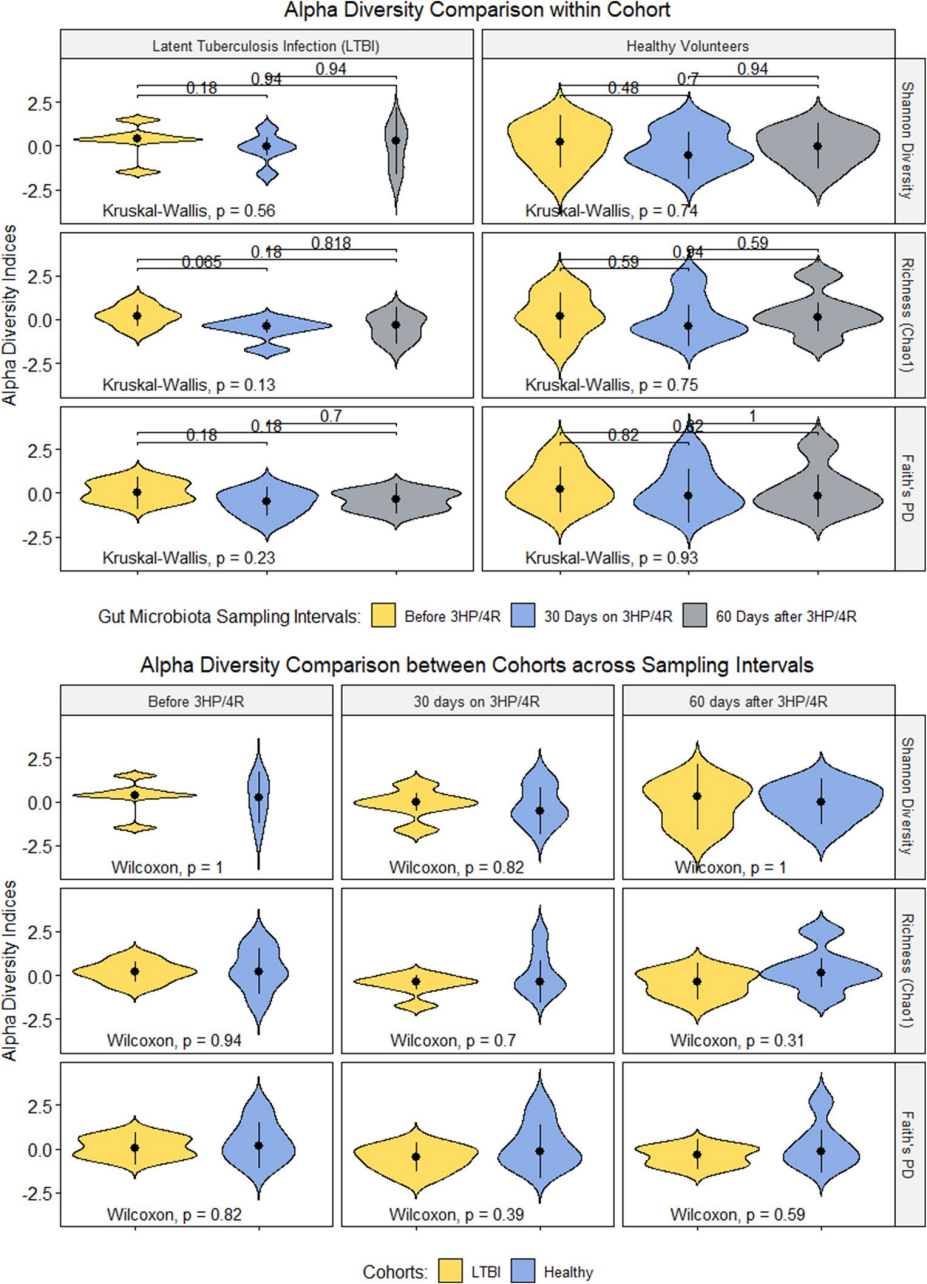
Gut microbiota alpha diversity comparison within (top panel) and between (bottom panel) LTBI and healthy volunteer cohorts using stool samples collected before, during, and two months after tuberculosis preventive therapy (TPT). Healthy volunteers did not receive TPT but collected stool samples in parallel with LTBI subjects on self-administered 4R. Each study participant collected three stool samples during TPT. Here we show the results of analyses conducted on the stool sample collected after 30 days on TPT. Results of analyses comparing alpha diversity across all five stool samples are shown in Supplementary Fig. 2. Our findings suggest a decrease in alpha diversity following rifamycin-based TPT initiation. The results did not vary when samples collected by participants who reported antibiotic and probiotic use during study follow-up were excluded.

To determine the immediate effect of rifamycin-based TPT on gut microbiota composition, we compared alpha diversity estimates for gut microbial species recovered in stool samples collected on days 0, 30, 60, and 90/120 of TPT initiation. The median and interquartile range of the Shannon, Chao1, and Faith’s Phylogenetic Diversity (PD) indices within and between cohorts across sampling interval are summarized in Fig. 1. The alpha diversity estimated from each of the five samples collected from each participant is shown in Supplementary Fig. 2. Because our cohorts were divers in age and baseline weight, we formally tested the influence of these variables on the baseline gut microbiota alpha diversity (Supplementary Fig. 3). Participant’s weight at baseline showed a weak positive correlation with Shannon Diversity but a weak negative correlation with Faith’s PD. Age explained a moderate fraction of the variance in alpha diversity at baseline, although the relationship did not reach statistical significance. In the healthy volunteer cohort, alpha diversity did not significantly change across the study observation period. We focused on the stool samples collected on day 30 of TPT to facilitate comparisons of the alpha diversity indices between the TPT and post-TPT time points. The median species diversity in the gut microbiota of LTBI subjects differed from that of healthy volunteers' pre-TPT, during TPT, or two months after TPT, although the difference did not reach statistical significance due to our small cohort sizes (Fig. 1). Additional analyses where we averaged the alpha diversity indices for the three samples taken during TPT to create one aggregate time point for the TPT interval comparable to the pre-TPT time point showed similar results. Four participants reported antibiotic (AL012, AL005, and HC004) and probiotic (HC004) use during follow-up. The alpha diversity indices before, during, and two months after TPT did not change significantly when the samples from these participants were dropped from analyses comparing baseline to TPT and post-TPT alpha diversity of the gut microbiota (Supplementary Fig. 4).

**Figure 2:**
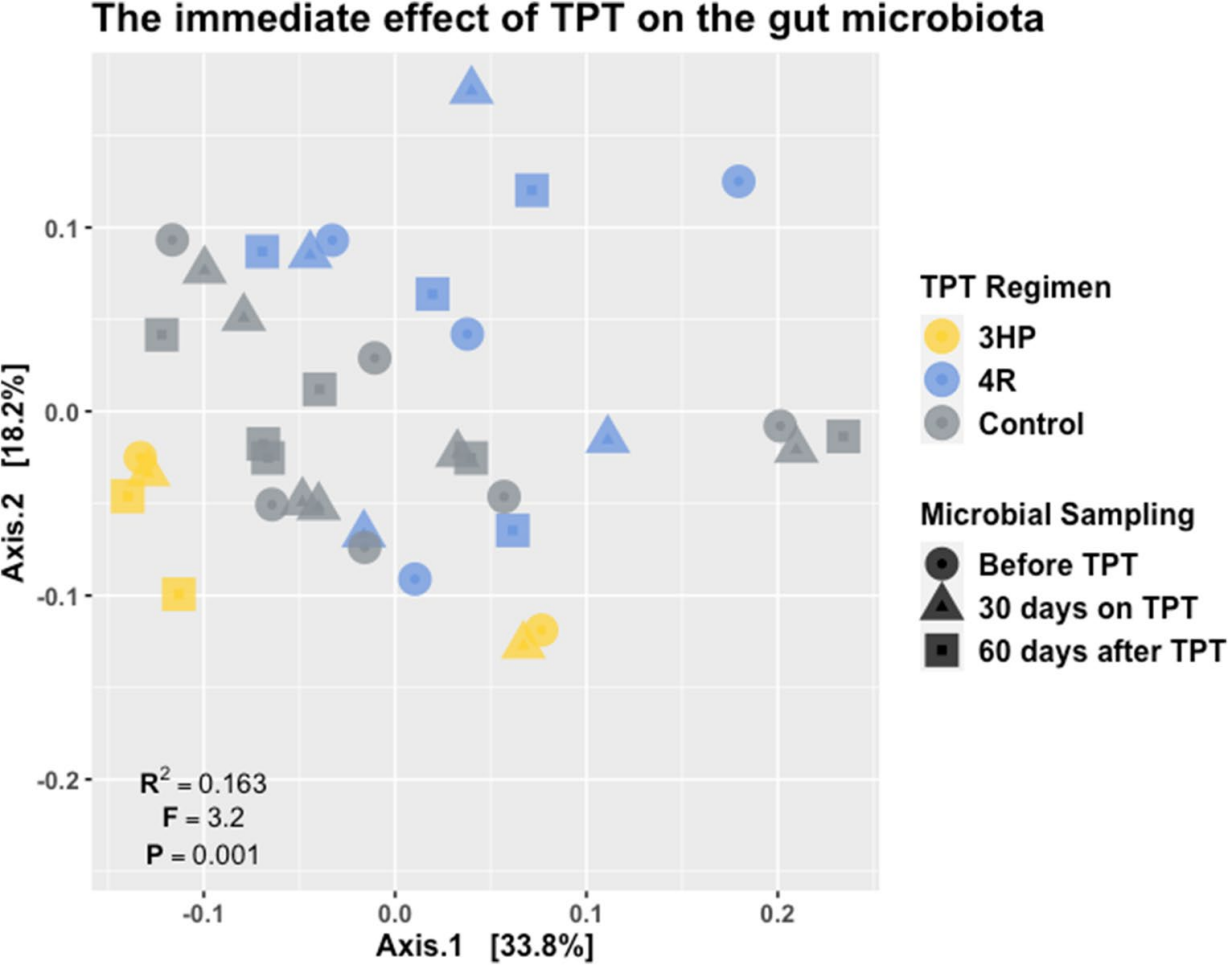
Principal coordinates analysis (PCoA) plot of the weighted UniFrac distance matrix calculated from amplicon sequence variants (ASV) randomly resampled to 33,582 sequence reads. For these analyses we focused on the first sample collected 30 days after TPT initiation. Pairwise comparison of the weighted UniFrac distances of the bacterial communities are mapped according to TPT exposure (color) and gut microbiota sampling intervals (shapes). Each point on the ordination represents a sample and points closer to each other suggest more similar bacterial communities while those further apart are more dissimilar. Permutational multivariable analysis of variance (PERMANOVA) results for the influence of rifamycin-based TPT on the gut microbiota community dissimilarity is shown on the plot (*R*^*2*^ = 0.163; *P* = *.001*). Overall, TPT regimen accounted for 17.0% of the variance in community dissimilarity. TPT with 3HP explained 10.5% (F = 13.2, *p* = 0.001) of the variance in community dissimilarity, while 6.5% (F = 8.2, *p* = 0.001) was explained by 4R.

We compared the weighted UniFrac distances between samples collected from LTBI and healthy volunteer cohorts separately to identify TPT-induced differences in gut microbiota community composition (Fig. 2). We observed a significant difference in the mean UniFrac distance between microbial communities sampled from the two cohorts (∆ mean UniFrac distance = 0.087, *p* = 0.024). TPT overall accounted for 17.0% (F = 4.5, *p* = 0.001) of the variance in community dissimilarity. 3HP dosed weekly for 3 months by DOT explained 10.5% (F = 13.2, *p* = 0.001) of the variance in community dissimilarity observed, while 4R dosed daily for four months by self-administration accounted for 6.5% (F = 8.2, *p* = 0.001) of the variance in weighted UniFrac distance. In contrast, we did not observe significant clustering in community dissimilarities in the healthy volunteer cohort (Supplementary Fig. 5; *R*^2^ = *0.021, P* = *1*). These observation suggests that bacterial community dissimilarity observed between the cohorts is due to rifamycin exposure and this TPT-induced dysbiosis is further modified by the treatment regimen. Except for the non-significant correlation between sampling interval and community dissimilarity, the results remained consistent when we repeated these analyses after removing observations from the two LTBI and two healthy volunteers who reported antibiotic and probiotic use during study follow (Supplementary Figs. 6–7). The relative abundance of eight bacterial taxa in the *Firmicutes* and *Proteobacteria* phyla were found to have changed following rifamycin exposure (Supplementary Table 3). Within the *Firmicutes* phylum, the counts of the genera *Clostridium_XIVa*, *Romboutsia, and Roseburia* were depleted during TPT compared to before TPT, while the abundance count of *Clostridiales*, *Coprococcus*, *Lachnospiraceae*, and *Ruminococcaceae* increased in numbers. The relative abundance of the genus *Parasutterella* within the phylum *Proteobacteria* also increased after exposure to the rifamycins.

### Intestinal microbiota recovery two months after tuberculosis preventive therapy

**Figure 3:**
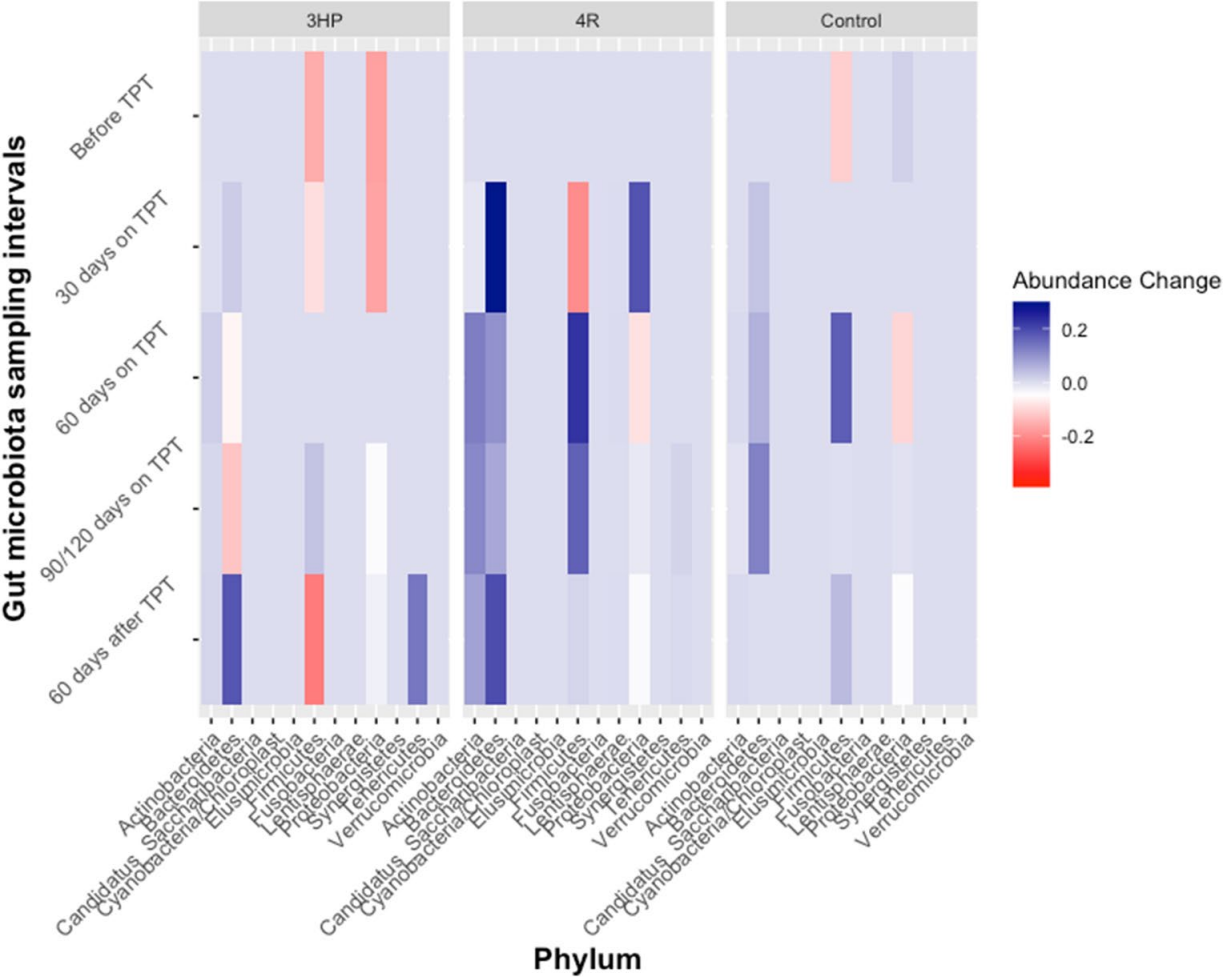
Magnitude and direction of TPT induced change in relative abundance of taxa aggregated at the phylum level. LTBI participants collected stool samples at home every 30 days, calculated from the date TPT was started. Here we show the change in relative abundance of each phylum compared to the relative abundance of phylum in samples collected before TPT. Healthy volunteers were not exposed to rifamycins but collected stool samples in parallel with LTBI subjects prescribed 4R.

We repeated the statistical analysis described above to investigate the extent to which the gut microbiota recovers to pre–TPT baseline. The alpha diversity indices increased two months after TPT, although the gut microbiota community diversity remains lower than observed pre-TPT. On the other hand, the alpha diversity indices of the gut microbiota of healthy volunteers remain stable across the different sampling intervals (Fig. 1). Since our cohort sizes are small and demographically diverse, and we observed a modest change in alpha diversity and UniFrac distance from baseline in both cohorts, we describe the magnitude and direction of phylum-level change in relative abundance for each study participant and summarize these data in Fig. 3. Statistical support for these comparisons is presented in Supplementary Fig. 8. Five phyla (*Candidatus_Saccaribacteria, Cyanobacteria/Chloroplast, Elusimicrobia, Fusobacteria,* and *Synergistetes)* remain unchanged across all sampling intervals in all participants. In four out of six LTBI volunteers, *Actinobacteria* increased in relative abundance two months after TPT, compared to their baseline relative abundance. The two LTBI participants (AL002 and AL007) in whom we observed a decrease in *Actinobacteria* from baseline were prescribed 4R by self-administration and reported gastrointestinal discomforts during therapy. AL007 disclosed taking probiotics to alleviate the gastrointestinal side effects but AL002 did not report changes to their behavior or diet during TPT. *Actinobacteria* decreased substantially in relative abundance for all healthy volunteers, except for two. HC001 reported a change in their diet during the TPT sampling intervals, and HC006 disclosed increased alcohol consumption during the post-TPT sampling interval. The phylum *Verrucomicrobia* decreased substantially in relative abundance in four LTBI subjects (AL003, AL005, AL012, MD001) but remained unchanged or increased in the healthy volunteers. We observed a two- and six-fold increase in the relative abundance of *Tenericutes* in the two LTBI subjects treated with 3HP by DOT (AL012 and AL002). A decrease in *Tenericutes* was observed in other LTBI participants, but the numbers were unchanged for HC002, HC003, HC005, and HC006, decreased by 100% in HC004 while increasing by 58% in HC001. Overall, some taxa depleted in numbers upon rifamycin exposure increased in relative abundance two months after TPT; however, the gut microbiota remained different from baseline. Two LTBI patients (AL002 and AL012) had trace concentrations of the parent rifamycin, and one patient (AL005) had trace concentrations of the partially active metabolite in the stool samples collected two months after TPT, which may explain the incomplete gut microbiota recovery to baseline (Supplementary Table 5).

## Discussion

Current anti-TB therapies (ATT) carry a significant risk of liver injury and neurological side effects^15^. Another potential important side effect of ATT could be long-term sequalae to the host microbiome or dysbiosis^20^. The gut microbiota plays a crucial role in health^38^. Crosstalk between the gut and lung microbiome via the gut-lung axis is hypothesized to support the host in fending off challenges by respiratory pathogens, such as Mtb^39^. ATT-induced disruption to the gut microbiota could potentially disrupt this important immune pathway. Our study is interested in the magnitude of dysbiosis induced by rifamycin-based preventive therapy (TPT) in persons at high-risk of TB disease and the extent to which the gut microbiota recovers from this disruption two months after TPT. People elect to undergo TPT to decrease their risk of TB disease progression. Thus, the intervention carries significant public health benefits^7^. Consequently, we were interested in the extent to which TPT results in gut microbiota changes that are significantly different from changes observed in a parallel cohort of individuals who are unexposed to the rifamycins under investigation. We recruited subjects initiating TPT from a representative population after they had accepted to be treated. The healthy volunteer cohort would have received the same diagnosis and treatment options had they been diagnosed with LTBI. We followed individuals in both cohorts prospectively to document immediate change to the gut microbiota following ingestion of the first rifamycin dose. Our findings suggest that the rifamycins induced a modest but significant shift in gut microbiota community dissimilarity in a pattern that was highly individualized. In some participants, the bacterial families Lachnospiraceae (Genus Coprococcus), Ruminococcaceae, and the genus Parasutterella increased in relative abundance during TPT, while in others the relative abundance of Roseburia (Family Lachnospiraceae) decreased during TPT. Treatment naïve TB patients have previously been shown to differ from healthy controls on the relative abundance of Ruminococcaceae and Lachnospiraceae, which are characterized by their anti-inflammatory properties as well as their ability to utilize carbohydrates in simple and polymeric forms to produce short-chain fatty acids (SCFAs) essential in the maintenance of health^40^. Although our numbers are too small to test this formally, prior reports suggest that a low level of Ruminococcaceae at baseline is associated with antibiotic-induced diarrhea^41^. Two LTBI subjects who reported gastrointestinal (GI) discomfort upon TPT initiation saw a resolution of their symptoms once treatment was completed.

We observed a shift in community composition consistent with prior reports from studies conducted in populations treated with a combination of INH, RIF, PZA, and ETH (HRZE) daily for a minimum of six months^20,42^. In our study, LTBI subjects received either 600 mg of RIF daily for four months by self-administration, or 900 mg of RPT and 600 mg of INH weekly DOT for three months. We observed that beta diversity was larger in LTBI subjects on 3HP compared to those receiving 4R, with 3HP accounting for a larger share of the variance in gut microbiota community dissimilarity observed in our study population. The differences in dosage and duration of 3HP and 4R may explain why 3HP was more disruptive to the gut microbiota than 4R. An alternative explanation is non-adherence to the 4R regimen administered SAT. In LTBI subjects treated by DOT, we observed a significant increase in the relative abundance of Tenericutes phylum, which includes genera lacking a peptidoglycan cell wall that are pathogenic to humans^43^. Tenericutes bacteria simple cell wall make them resistant to the ATT drugs which target the mycolic acid cell wall structure of Mtb.

One of our objectives was to document recovery of the gut microbiota following TPT. We provide preliminary evidence that the rifamycin-induced dysbiosis to the gut microbiota is partially reversible two months after TPT. However, our findings are not conclusive, and should be evaluated with caution, considering that this was an observational study, and participants were divers in demographic characteristics known to confound results of human microbiota studies^44^. We followed our study participants through the Thanksgiving and Christmas holidays and the first few weeks of stay-at-home orders to control COVID-19 spread. Participants reported eating and drinking alcohol more heavily during these times. LTBI volunteers were counselled by the TB clinic to avoid alcohol during TPT to limit liver injury^45^. However, healthy volunteers were not instructed to modify their diet or lifestyle. Alcohol and other drastic diet changes have been shown to alter the composition and diversity of the gut microbiota^46,47^. A similar effect in this study may have contributed to the healthy volunteers’ gut microbiota resembling more the gut microbiota of the treated LTBI subjects, thus resulting in a smaller overall impact of the rifamycin exposure. 

We discontinued follow-up two months after TPT, which likely was too short of a follow-up time to document complete reversal to baseline for all LTBI patients^48,49^. In particular, three out of six LTBI patients had trace concentrations of the parent rifamycins or their partially active metabolites in the stool samples collected two months after taking their last treatment dose. The rifamycins are primarily eliminated in the stool and to a lesser extent in urine^15^. In these LTBI participants, our estimate of the post-TPT recovery of the gut microbiota is confounded by the delayed clearance of the rifamycins. In addition, one LTBI participant reported antibiotic use during the post-TPT follow-up period which further confounded our estimates of the gut microbial reversal to baseline for one LTBI participant. Finally, the choice of the hypervariable region of the 16S rRNA used to profile the gut microbiota is a major influence on species representation, taxa richness, and diversity^50^. We aimed to measure the immediate change in gut microbiota composition and diversity following exposure to 3HP or 4R by profiling the gut microbiota at 30-day interval during TPT and then sixty days after TPT. We believe the V1-V2 region of the 16S rRNA encompasses sufficient phylogenetic resolution to address our study objectives. Nevertheless, bias due to under-sampling of the gut microbiota cannot be eliminated.

In conclusion, important literature on the synergistic interplay between the human gut and lung microbiome on the prevention and control of TB is slowly emerging, but questions remain. Our study adds to this literature by prospectively tracking the effect of TPT with 3HP or 4R on the diversity and composition of the gut microbiota before, during and two months after therapy in the same individuals. Overall, our small study provides an initial look into the magnitude of gut dysbiosis induced by rifamycin-based TPT to motivate interest into the potential long-term sequalae to the host microbiome of this important public health function to eliminate TB in low incidence settings.

### Supplementary Information

Supplementary Information.

## Data Availability

The ASVs and associated clinical data described here can be accessed on figshare.com (10.6084/m9.figshare.21381930.v1). The raw amplicon sequences can be accessed on NCBI under accession number PRJNA772261.

## Abbreviations

3HP: Three months of weekly isoniazid and rifapentine
4R: Four months of daily rifampin
ASV: Amplicon sequence variant
DNA: Deoxyribonucleic acid
EPI: Emerging pathogens institute
HIV: Human immunodeficiency virus
IDPL: Infectious disease pharmacokinetics laboratory
IGRA: Interferon gamma release assay
INH: Isoniazid
LC–MS-MS: Liquid chromatography-tandem mass spectrometry
LTBI: Latent tuberculosis infection
Mtb: *Mycobacterium tuberculosis*
PLWH: People living with HIV
RIF: Rifampin
RNA: Ribonucleic acid
RPT: Rifapentine
rRNA: Ribosomal ribonucleic acid
TB: Tuberculosis
TBI: Tuberculosis infection
TPT: Tuberculosis preventive therapy
TST: Tuberculin skin test
