# Supplementary material for: Gut microbiota composition and diversity before, during, and two months after rifamycin-based tuberculosis preventive therapy
Source: Sci Rep. 2023 Nov 2;13:18933. doi: 10.1038/s41598-023-44854-5 (PMC10622450; doi:10.1038/s41598-023-44854-5)
Supplement: Supplementary file 1 — Supplementary Information. [file 41598_2023_44854_MOESM1_ESM.docx]

Supplementary Material

Gut Microbiota Composition and Diversity Before, During, and Two months after Rifamycin-based Tuberculosis Preventive Therapy

Marie Nancy Séraphin^1,2*^, Julia Bellot^1,2^, Emily Klann^,2,3^, Maria Ukhanova^2,3^, Florence G. Saulsberry^4^, Charles A. Peloquin^2,5^, and Volker Mai^2,3^

^1^Department of Medicine, Division of Infectious Diseases and Global Medicine, College of Medicine, University of Florida, Gainesville, Florida, United States

^2^Emerging Pathogens Institute, University of Florida, Gainesville, Florida, United States

^3^Department of Epidemiology, College of Public Health and Health Professions and College of Medicine, University of Florida, Gainesville, Florida, United States

^4^Florida Department of Health in Alachua County, Disease Control Unit, Alachua County Health Department, Gainesville, Florida, United States

^5^Infectious Disease Pharmacokinetics Laboratory, College of Pharmacy, University of Florida, Gainesville, Florida, United States

*** Correspondence:**Marie Nancy Séraphin
[nseraphin@ufl.edu](mailto:nseraphin@ufl.edu)

**Keywords**: latent tuberculosis infection, 3HP, 4R, rifamycins, gut microbiome, dysbiosis, tuberculosis preventive therapy

# Methods

## *Ethical Clearance*

The study protocol was reviewed and approved by the University of Florida (IRB201801385) and the Florida Department of Health (2018-057) Institutional Review Boards, respectively. Each participant provided written informed consent before all study activity. Participants received $125 in incentive; $25 for each stool sample provided.

## *Stool collection at home*

At enrollment, all participants received a kit that included a stool collection bucket, a 5ml screw top tube containing 3ml of RNA*later*™ Stabilization Solution (AM7021), three ceramic beads (2.8 mm), a wooden stick, and instructions to collect about 1g of stool at home and ship to the University of Florida Emerging Pathogens Institute (EPI) in a prepaid bubble mailer, where they were immediately stored at -80^0^C.

## *DNA extraction and 16S rRNA amplicon sequencing*

Stool samples were batched for DNA extraction. On extraction day, the samples were thawed at room temperature then kept on ice during processing. The color and consistency of each stool sample were recorded before proceeding with bacterial genomic DNA isolation using a modified Qiagen fecal DNA extraction protocol with initial mechanical lysis bead-beating step (Biospec Products), as previously described^1^. Procedures to profile the gut microbiota via amplification of the V1-V2 hypervariable regions of the bacterial 16S rRNA were performed at the EPI following standard protocols as previously described^2^. Equimolar amounts of the 16S libraries were pooled for paired-end sequencing (250 x 2) using the Illumina Mi-Seq platform (Illumina®, San Diego, CA).

## *Parent and Metabolite drug concentration measurement*

The DNA extraction protocol was modified to recover the supernatant from the initial phosphate buffer wash of the stool samples. Briefly, all samples were homogenized by vortexing, and 0.75mL was transferred to a bead beater tube. We added 1mL of phosphate buffer and vortexed quickly to mix. The samples were then centrifuged for 5:00 minutes at 15.4 RCF. The supernatant from this initial wash step was transferred to labeled 2mL tubes and stored at -80^o^ C until drug concentration assays were performed using standard procedures developed in the UF Infectious Disease Pharmacokinetics Laboratory (IDPL). A modification of the IDPL assay was used to measure the concentrations of the parent rifampin, rifapentine, and their major metabolite, desacetylrifampicin and 25-O-desacetylrifapentine, respectively in PBS wash instead of plasma, using a validated liquid chromatography-tandem mass spectrometry (LC-MS-MS) assay on a Thermo Acella high-performance liquid chromatography system and a Thermo Ultra triple-quadrupole mass spectrometer with a Dell computer and the Thermo Xcaliburdata management system.

# Supplementary Figures and Tables

## Supplementary Figures

##
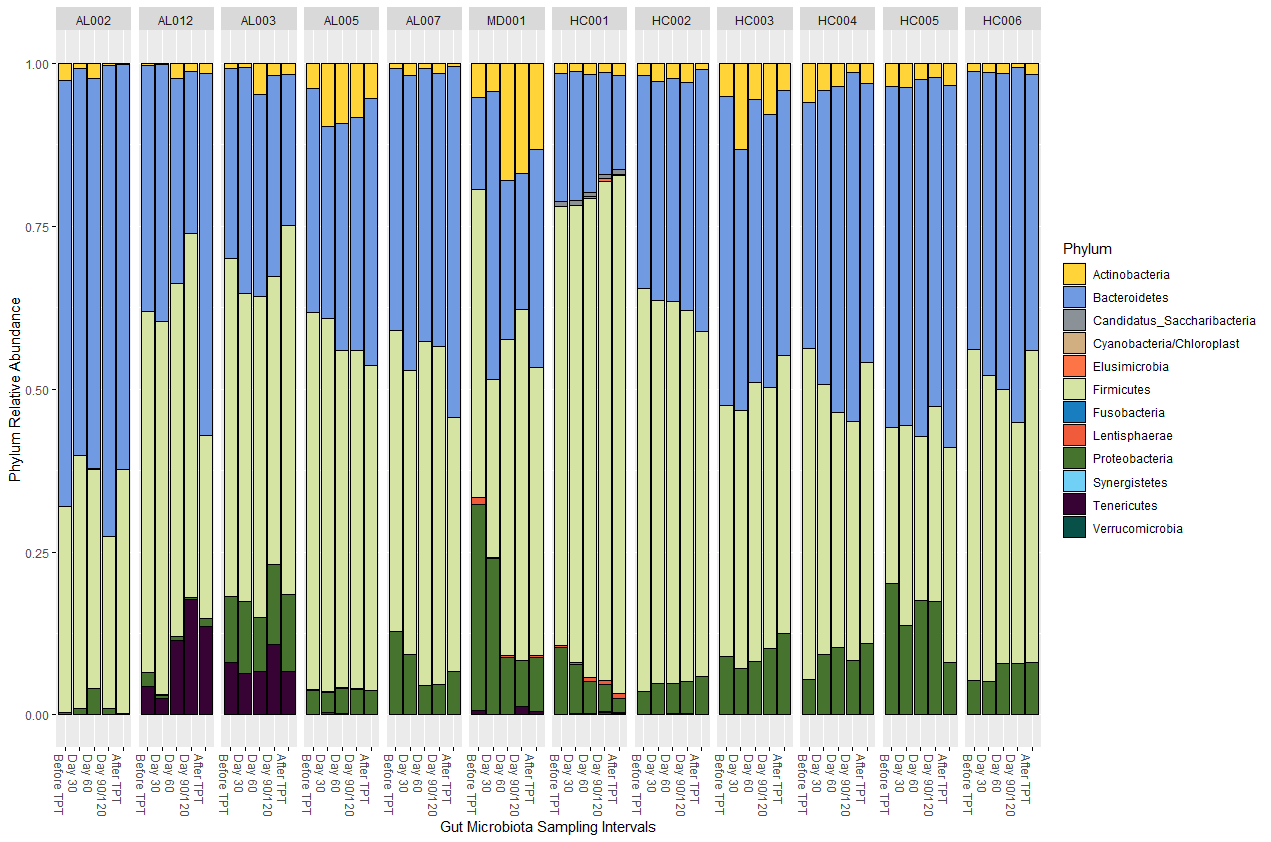
Supplementary Figure 1. Phylum relative abundance estimated as a proportion of the total number of amplicon sequence variants (ASV) in each participant sample. Sequencing reads were randomly resampled to 33,582 to ensure even sampling of the gut microbiota. The figure shows phylum relative abundance in stool samples collected by LTBI (AL002 – MD001) and healthy volunteers (HC001 – HC006) and ordered by sampling interval. Healthy volunteers were unexposed to rifamycins but collected stool samples in parallel with LTBI subjects exposed to 4R. All study participants had not used antibiotics at least one month prior to study enrollment. AL005, AL012, and HC004 reported the use of antibiotics during follow-up (second and post-TPT intervals), while AL007 introduced daily probiotics during TPT (first and second sampling intervals).

**
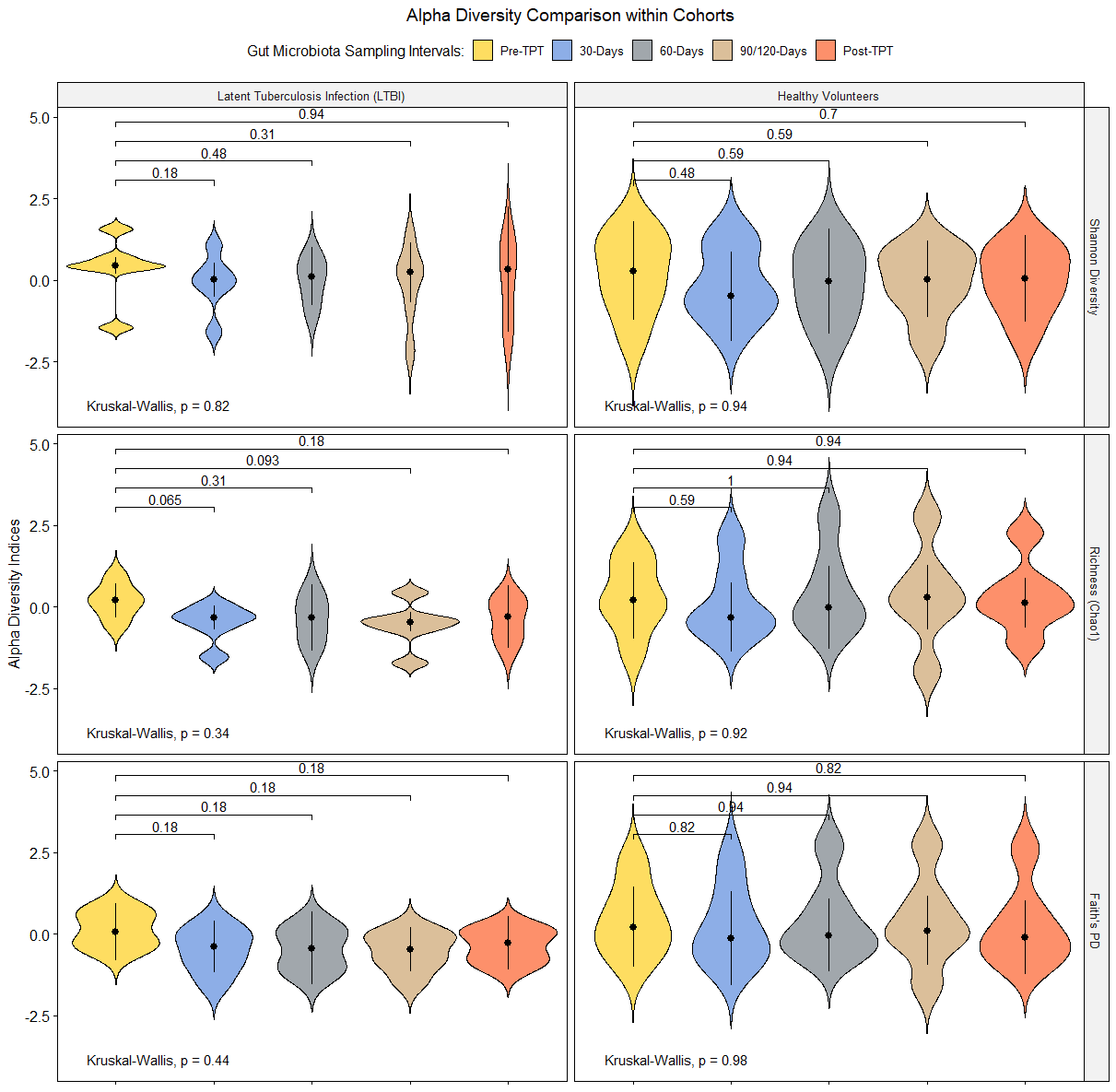
Supplementary Figure 2**. **Alpha diversity of the gut microbial community of LTBI and healthy volunteer cohorts before, during and after rifamycin-based tuberculosis preventive therapy**. Healthy volunteers did not receive rifamycin-based tuberculosis prevention therapy (TPT) but collected stool samples at concurrent intervals as the LTBI patients treated with 4R. alpha diversity decreased in the 30 days after TPT initiation compared to baseline, although this difference did not reach statistical significance due to our sample size. In comparison, alpha diversity in the healthy volunteers remained stable over the study duration.

**
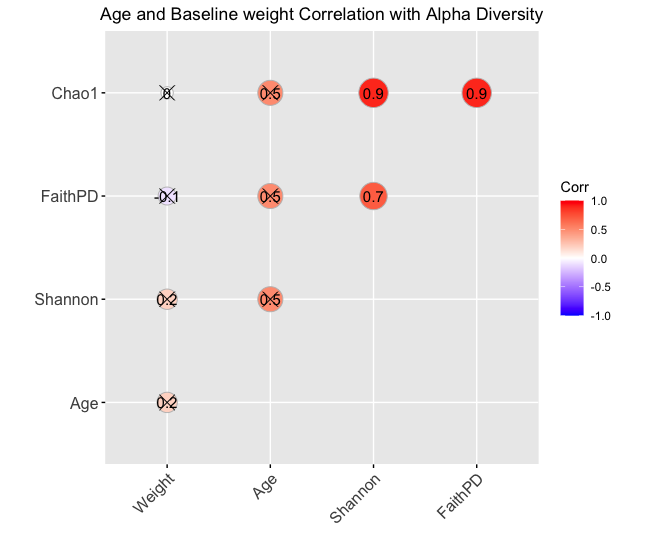
**

Supplementary Figure 3. Age and baseline weight correlation with alpha diversity and richness. Baseline weight shows a weak positive correlation with Shannon diversity and a weak negative correlation with Faith’s PD. Age explained a moderate fraction of the variance in alpha diversity and richness at baseline, although the relationship did not reach statistical significance.


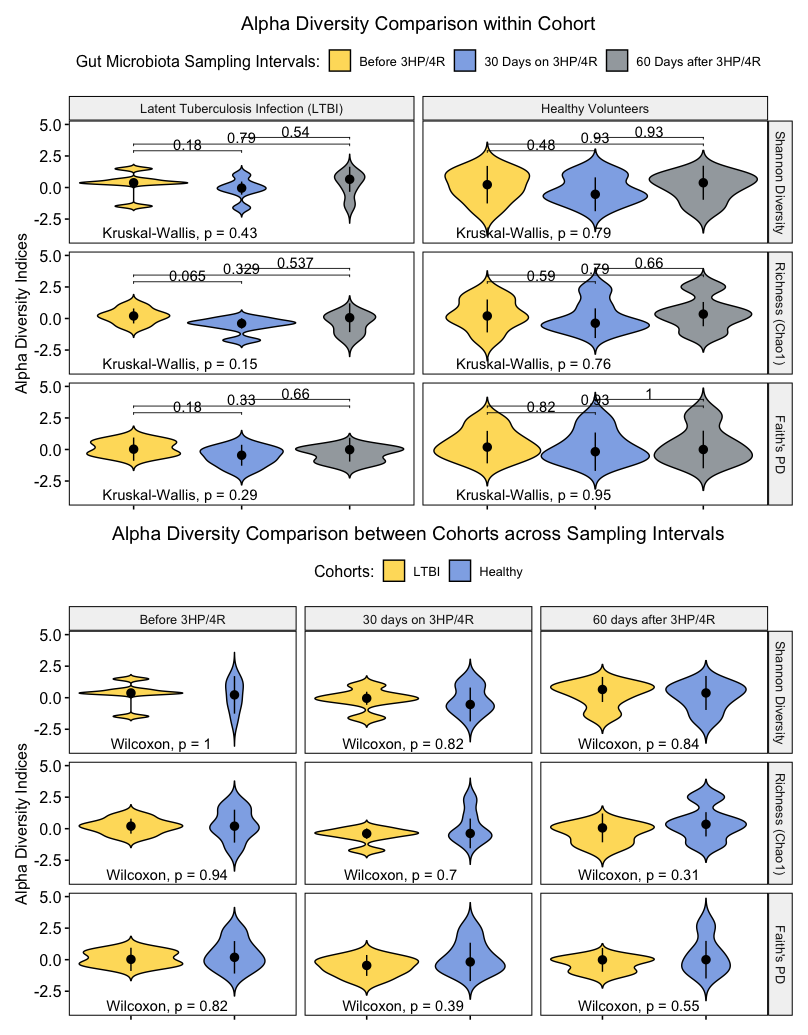


Supplementary Figure 4. Alpha diversity comparison within (top) and between (bottom) LTBI and healthy volunteer cohorts before, during, and two months after tuberculosis preventive therapy (TPT). The figure shows the data presented in main manuscript Figure 1. Here, we dropped observations from participants who reported antibiotic and probiotic use during study follow up. Specifically, these samples were excluded: AL005-2, AL012-4, AL007-1, AL007-2, and HC004-4. We did not observe a significant change in our measurement of the rifamycin-based TPT-induced change in alpha diversity as a result of dropping these samples from the analysis.


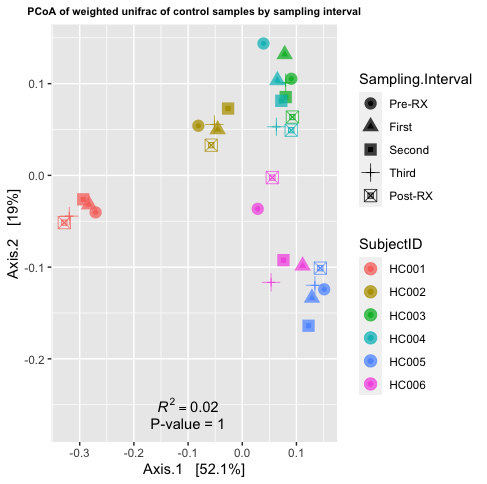


**Supplementary Figure 5.** **Principal coordinates analysis (PCoA) plot on the weighted UniFrac distance matrix generated from ASVs randomly resampled to 33,582 sequence reads and depicting patterns of beta diversity for gut microbial communities of healthy volunteers**. Points represent the beta diversity at a sampling interval for each of the six participants. Each healthy volunteer provided three stool samples during treatment (First, Second, Third) collected at 30, 60, and 180 days from baseline. Points that are closer together on the ordination have more similar bacterial communities. Permutational multivariate analysis of variance analysis indicates that differences between microbial communities across sampling intervals are not significantly different (*R*^2^ = 0.02; *P = 1*). These analyses were repeated to exclude the post-TPT sample collected by HC004 who reported antibiotics use, without significant changes to the results.

Supplementary Figure 6. Principal coordinates analysis (PCoA) plot on the weighted UniFrac distance matrix after removing observations from participants who reported using antibiotics during treatment (AL005 – Second sampling interval) and posttreatment (AL012, HC004 – Post-RX). Observations from AL007 who reported probiotic use at months one (First) and two (Second) of their 4R regimen were also excluded. Points closer to the ordination have more similar bacterial communities. Left panel depicts patterns of beta diversity for bacterial communities sampled from LTBI patients only. Permutational multivariate analysis of variance analysis indicates that differences between communities mapped according to individual patients (colors) and sampling interval (shapes) are non-significant (*R^2^ =* 0.067; *P = .379*). Right panel depicting the patterns of beta diversity for bacterial communities sampled from LTBI and controls shows a significant PERMANOVA for the differences between communities mapped according to treatment [(3HP, 4R, or control) (*R*^2^ = 0.152*; P = .001*)]


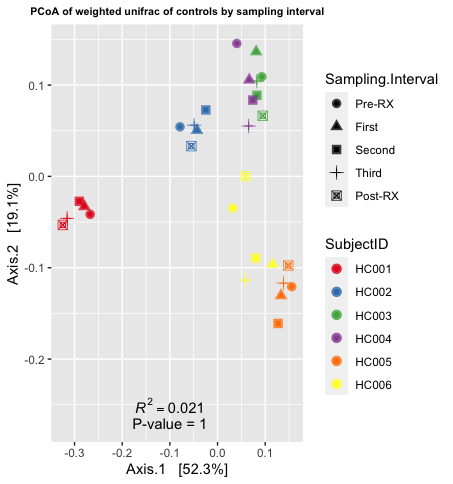


**Supplementary Figure 7**. Principal coordinates analysis (PCoA) plot on the weighted UniFrac distance matrix generated from ASVs randomly resampled to 33,582 sequence reads and depicting patterns of beta diversity for gut microbial communities of healthy volunteers. Points represent the beta diversity at a sampling interval for each of the six participants. The posttreatment sample collected from HC004 was dropped from these analyses, as the participant reported taking antibiotics. Points that are closer together on the ordination have more similar bacterial communities. Permutational multivariate analysis of variance analysis indicates that differences between microbial communities across sampling intervals are not significantly different (*R*^2^ = 0.021; *P = 1*).


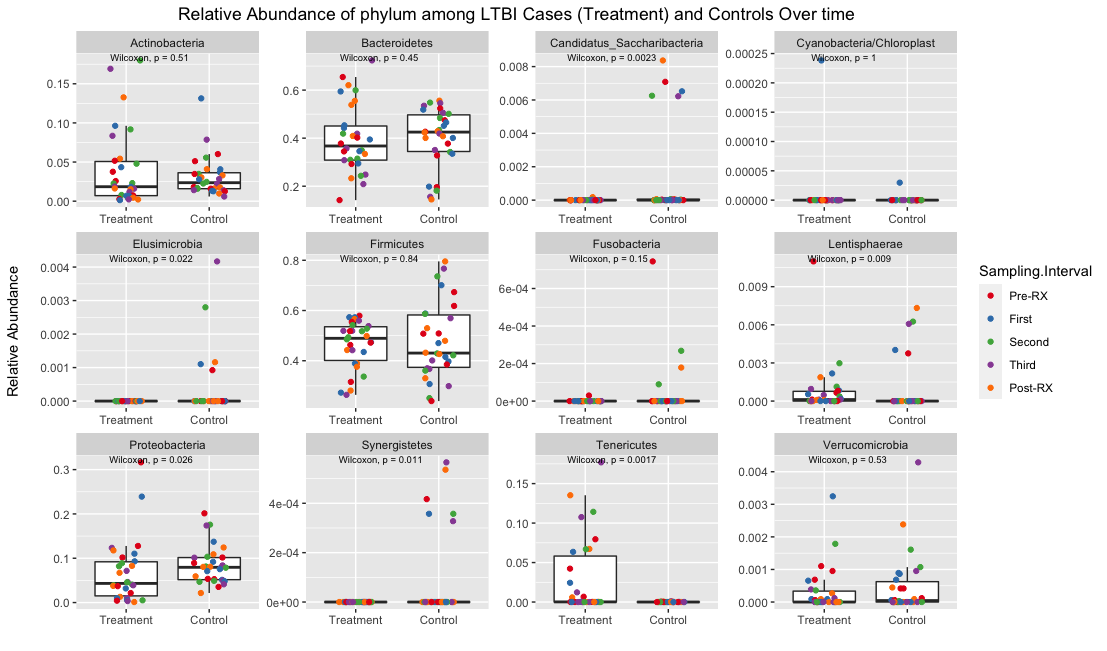


**Supplementary Figure 8.** **The relative abundance of bacterial taxa aggregated at the phylum level in LTBI and healthy volunteers cohorts**. Each dot represents one of five samples collected from each participant. Dot color indicates the sampling timepoint for each sample. Significant differences in relative abundance of *Candidatus_Saccharibacteria*, *Elusimicrovia*, *Lentisphaerae*, *Proteobacteria*, *Synergistetes*, and *Tenericutes* were observed between LTBI patients and controls.

## Supplementary Tables

**Supplementary Table 1. Timing of stool sample collection by each study participant**

| **Participant**  **(TPT regimen)** | **Enrollment to baseline** | **Before TPT sample** | **Day 30 of TPT Sample** | **Day 60 of TPT Sample** | **Day 90/120 of TPT Sample** | **Day 60 After TPT sample** |
| --- | --- | --- | --- | --- | --- | --- |
| AL002 (3HP) | 6 | 8 | 36 | 82 | 100 | 156 |
| AL003 (4R) | 1 | 0 | 30 | 61 | 122 | 179 |
| AL005 (4R) | 0 | 0 | 31 | 60 | 122 | 178 |
| AL007 (4R) | 1 | 0 | 28 | 67 | 99 | 182 |
| AL012 (3HP) | 0 | 0 | 31 | 64 | 78 | 150 |
| MD001 (4R) | 0 | 0 | 21 | 51 | 110 | 170 |
| HC001 | 0 | 0 | 30 | 63 | 119 | 179 |
| HC002 | 5 | 0 | 31 | 56 | 123 | 182 |
| HC003 | 1 | 0 | 35 | 60 | 122 | 180 |
| HC004 | 29 | 0 | 33 | 62 | 120 | 180 |
| HC005 | 2 | 0 | 28 | 66 | 122 | 180 |
| HC006 | 1 | 0 | 32 | 58 | 120 | 180 |

**Notes**: Stool samples during tuberculosis preventive therapy (TPT) were collected at 30-day interval ±7 days, estimated based on the date the LTBI participants ingested their first dose of the rifamycin regimen. We fixed the “treatment start date” for healthy volunteers using the date the baseline sample was collected. 3HP was by direct observation at the TB clinic or via video while 4R was by self-administration.

**Supplementary Table 2. Change in taxa abundance during and two months after, compared to before rifamycin-based tuberculosis preventive therapy (TPT)**

|  | **Abundance count** | | |  |  |
| --- | --- | --- | --- | --- | --- |
| **Bacterial taxa (ASV)** | **Before TPT** | **During TPT** | **After TPT** | **TPT induced changes**  **(P-value) *** | **After TPT Recovery**  **(P-value) *** |
| Clostridiales | 0 | 34 | - | 0.074 |  |
| Clostridium_XIVa | 201 | 62 | 104 | **0.002** | 0.065 |
| Coprococcus | 0 | 6 | - | 0.074 |  |
| Lachnospiraceae****** | 43 | 118 | 0 | 0.087 | 0.074 |
| Parasutterella | 0 | 31 | - | 0.074 |  |
| Oscillibacter | 43 | - | 0 |  | 0.074 |
| Romboutsia | 18 | 0 | 0 | 0.074 | 0.074 |
| Roseburia** | 75 | 0 | - | **0.028** |  |
| Ruminococcaceae | 0 | 29 | - | **0.015** |  |

**Notes:** *P-values were calculated using the non-parametric paired Wilcoxon test and were corrected using the Benjamini-Hochberg method. Some p-values are similar as the wilcox.test function in R returns normal approximated p-values for less than 50 observations with ties. Only ASVs associated with a p-value < 0.1 are included in this table. **Multiple ASVs classified as Lachnospiraceae (N=2) and Roseburia (N=2) were identified as differentially abundant. Only the ASVs with the largest differential abundance are shown here. At the exception of Parasutterella, all bacterial taxa shown group under the phylum Firmicutes. The data were rarefied to diminish differences due to sequence depth prior to conducting these analyses. Three samples were collected from each participant during the treatment interval. In order to compare this interval to the pre-treatment interval with only one sample, we pooled the three samples from each participant.

Supplemental Table 3. Parent and metabolite rifamycin concentrations and their ratios (Cm/Cp) recovered in stool samples collected during treatment and two months after treatment completion.

|  | **30 days on TPT** | | | **60 days on TPT** | | | **90/120 days on TPT** | | | **60 days after TPT** | | |
| --- | --- | --- | --- | --- | --- | --- | --- | --- | --- | --- | --- | --- |
| **Subject ID** | **Parent Rifamycin** | **Metabolite** | **Cm/Cp*** | **Parent Rifamycin** | **Metabolite** | **Cm/Cp*** | **Parent Rifamycin** | **Metabolite** | **Cm/Cp*** | **Parent Rifamycin** | **Metabolite** | **Cm/Cp*** |
| AL002 | 0.422 | 0.093 | 0.220 | 0.011 | 0.010 | 0.895 | 0.011 | 0.012 | 1.031 | 0.008 | 0.002 | 0.218 |
| AL003 | 9.593 | 13.024 | 1.358 | 4.404 | 3.354 | 0.762 | 0.233 | 0.292 | 1.255 | 0.001 | 0.000 | 0.162 |
| AL005 | 9.720 | 37.401 | 3.848 | 1.954 | 14.062 | 7.195 | 4.850 | 19.470 | 4.014 | 0.001 | 0.015 | 10.488 |
| AL007 | 5.874 | 7.249 | 1.234 | 0.000 | 0.002 | 13.454 | 0.001 | 0.001 | 0.735 | 0.001 | 0.002 | 1.270 |
| AL012 | 0.087 | 0.001 | 0.009 | 2.614 | 0.094 | 0.036 | 0.765 | 0.012 | 0.016 | 0.010 | 0.001 | 0.061 |
| MD001 | 8.268 | 28.834 | 3.487 | 14.287 | 68.097 | 4.766 | 1.394 | 4.014 | 2.88 | 0.001 | 0.001 | 2.811 |

**Notes**: *Cm: rifamycin metabolite concentration; Cp: parent rifamycin metabolite concentration. Cm/Cp measures the ratio of parent drug to metabolite that was recovered in stool. To avoid dividing by zero some noise was added to the data using the base:jitter function in R. Drug concentration was measured at UF - Infectious Disease Pharmacokinetics Laboratory (IDPL), using an assay modified to measure the rifamycins parent and metabolite concentrations in a phosphate buffer wash of the stool samples collected during TPT and two months after TPT in the LTBI cohort.

**References**

1. Yaghjyan, L. *et al.* Gut microbiome, body weight, and mammographic breast density in healthy postmenopausal women. *Cancer Causes Control CCC* **32**, 681–692 (2021).

2. Klann, E. *et al.* Microbiota composition in bilateral healthy breast tissue and breast tumors. *Cancer Causes Control* **31**, 1027–1038 (2020).
